# Cytotoxicity of Mycotoxins Frequently Present in Aquafeeds to the Fish Cell Line RTGill-W1

**DOI:** 10.3390/toxins13080581

**Published:** 2021-08-20

**Authors:** Elena Bernal-Algaba, Marta Pulgarín-Alfaro, María Luisa Fernández-Cruz

**Affiliations:** Department of Environment and Agronomy, National Institute of Agriculture and Food Research and Technology (INIA), Spanish National Research Council (CSIC), 28040 Madrid, Spain; elena.bernal@inia.es (E.B.-A.); mpulgarin@cnic.es (M.P.-A.)

**Keywords:** mycotoxins, cytotoxicity, cell viability, RTgill-W1, fish

## Abstract

In the last decades, the aquaculture industry has introduced plant-based ingredients as a source of protein in aquafeeds. This has led to mycotoxin contaminations, representing an ecological, health and economic problem. The aim of this study was to determine in the RTgill-W1 fish cell line the toxicity of fifteen mycotoxins of common occurrence in aquafeeds. To identify the most sensitive endpoint of toxicity, the triple assay was used. It consisted of three assays: alamarBlue, Neutral Red Uptake and CFDA-AM, which revealed the mitochondrial activity, the lysosomal integrity and the plasma membrane integrity, respectively. Most of the assayed mycotoxins were toxic predominantly at lysosomal level (enniatins, beauvericin, zearalenone, ochratoxin A, deoxynivalenol (DON) and its acetylated metabolites 15-O-acetyl-DON and 3-acetyl-DON). Aflatoxins B1 and B2 exerted the greatest effects at mitochondrial level, while fumonisins B1 and B2 and nivalenol were not toxic up to 100 µg/mL. In general, low toxicity was observed at plasma membrane level. The vast majority of the mycotoxins assayed exerted a pronounced acute effect in the fish RTgill-W1 cell line, emphasizing the need for further studies to ascertain the impact of mycotoxin contamination of fish feeds in the aquaculture industry and to establish safe limits in aquafeeds.

## 1. Introduction

The worldwide human population has experienced significant growth in the last decades, leading to an increasing demand for fish as an accessible protein source for human consumption [1,2,3]. Simultaneously, aquaculture has been extensively developed to meet the challenge of feeding this expanding population. One of the most important points concerning the rapid development of aquaculture is the need for aquafeeds obtained from raw materials with certain requirements: (i) affordable, since a large amount of feed must be produced; (ii) with optimal nutritional features, in order to not compromise health, growth and reproductive performance; (iii) and safe, for both animals and humans [4]. One of the strategies followed to meet the first requirement has been the replacement of animal proteins with vegetable proteins, introducing cereals or legumes into feed composition [4,5,6].

The increased use of aquafeeds that are manufactured from vegetal raw materials has given rise to new concerns regarding aquaculture. Mycotoxin contamination has gained attention in this field due to its presence in both starting materials and finished feedstuffs [1,6,7]. Mycotoxins are toxic secondary metabolites produced by certain species of filamentous fungi that exhibit adverse effects in human and animals, leading to the development of various pathological conditions known as mycotoxicosis [1,8]. These conditions are very dissimilar in symptoms and severity, ranging from carcinogenicity to neurotoxicity, developmental toxicity and metabolic and reproductive disorders [1,4].

Mycotoxins are most commonly produced by species of the genera *Aspergillus*, *Fusarium* and *Penicillium* [9]. *Fusarium* species produce a wide range of mycotoxins, such as enniatins (ENNs), fumonisins (FBs), zearalenone (ZEN) and trichothecenes, a family of mycotoxins that include deoxynivalenol (DON), nivalenol (NIV) and T-2 and HT-2 toxins. Ochratoxin A (OTA), another prevalent mycotoxin in feeds and foods, is produced by different *Aspergillus* and *Penicillium* species, while aflatoxins (AFs) are mainly produced by *Aspergillus*. AFs are considered among the most dangerous mycotoxins from the point of view of food security due to their potent genotoxic and carcinogenic effects, with the liver as the main organ affected [10]. Mycotoxin contamination of aquafeeds containing plant-based materials can occur at different time points, both at pre-harvest (poor harvesting practices, nutrient availability, climatic conditions, etc.) and post-harvest (improper storage conditions or inappropriate crop handling and processing) stages. In addition, the type and the amount of mycotoxins present in feeds are highly dependent on the geographical location of the harvest, since this determines the starting materials that are used, the type of farming and other environmental factors, such as climate [4,11]. As reviewed by Pinotti et al. [11], certain types of mycotoxins, such as DON, ZEN and FBs, are geographically widespread. In contrast, other mycotoxins, such as OTA and AFs, are more restricted to geographical areas with hot, humid c1imates, such as Southern Europe, Africa and South and Southeast Asia [4,11]. Regarding the amounts of mycotoxins in feed materials, as surveyed worldwide by Marquardt and Madhyastha [12], FB1 was globally the most abundant mycotoxin (in ppb), followed by DON and ZEN. However, in the recent BIOMIN Mycotoxin Survey (https://www.biomin.net/solutions/mycotoxin-survey/, (accessed on 19 August 2021)), the most prevalent mycotoxins globally in 2020 were DON (65%), followed by FBs (64%) and ZEN (48%). Other mycotoxins that were found in this mycotoxin survey were the so-called emerging mycotoxins, such as moniliformin (56%), beauvericin (BEA; 48%), enniatin B1 (ENNB1; 35%), enniatin A1 (ENNA1; 28%), enniatin A (ENNA; 15%) and enniatin B (ENNB; 23%). Co-occurrence of different mycotoxins in crops and feeds is also common, raising additional concerns about potential combined or even synergistic toxic effects, as evidenced in vitro and in vivo [13]. In fact, among all analyzed samples in the BIOMIN Mycotoxin Survey, co-occurrence was detected in 53% of the samples, while 17% were free of mycotoxins (with levels below the limit of detection) and 30% were contaminated with a single mycotoxin.

The observed incidence and distribution of mycotoxins in aquafeeds indicate the need to assess their toxic effects in fish. In addition, mycotoxins present in fish feed might pass through the food chain and reach the consumer, also representing a health risk. In the aquaculture field, economic losses related to fungal and mycotoxin contamination were reported, and were due to an increase in the mortality of farmed fish as well as to a decrease in growth and reproductive output [1]. The toxicity of mycotoxins in fish has been progressively studied, although most of the studies were performed after chronic exposure and only a few have assessed the acute toxicity.

In the present study, a cell line from rainbow trout gill (RTgill-W1) was exposed over 24 h to 15 different mycotoxins, including some of the mentioned emerging mycotoxins, in order to assess their cytotoxic effects. These mycotoxins were selected because they have been reported as common aquafeed contaminants. The mycotoxins selected consist of four types of ENN (ENNA, ENNA1, ENNB and ENNB1), two types of FBs (FB1 and FB2), two types of AFs (AFB1 and AFB2), as well as ZEN, OTA, BEA, NIV and DON and two of its acetylated derivatives, 15-O-acetyl-4-DON and 3-acetyl-DON. This fish cell line was chosen as an alternative system to animal models to predict acute toxicity in fish, given that various studies support its suitability for this purpose [14,15]. Indeed, very recently a new OECD test guideline was published, the TG No. 249 “Fish Cell Line Acute Toxicity: The RTgill-W1 cell line assay” [16]. This test guideline was designed to predict fish acute toxicity in product testing.

## 2. Results

The cytotoxic effects associated with different mycotoxins were determined in the RTgill-W1 cell line through the assessment of toxicity at mitochondrial level by the alamarBlue assay, at plasma membrane level by the carboxyfluorescein diacetate-acetoxymethyl ester (CFDA-AM) assay, and at lysosomal level by the neutral red uptake assay (NRU). The three assays were conducted to identify the most sensitive endpoint of toxicity in order to avoid subestimations of toxicity and to explore possible mechanisms of cellular toxicity. Cell viability was determined after 24 h of treatment with a range of mycotoxin concentrations. The effective concentrations that decreased mitochondrial activity or plasma or lysosomal membrane integrities in 50% of the cells (EC_50_) were calculated for each assay (Table 1). These values indicated a high toxicity for the ENNs, BEA and ZEN, which showed a greater effect at the lysosomal level with EC_50_ ranging from 2.89 to 8.02 µg/mL, the ENNB being the least toxic (EC_50_ of 17.03 µg/mL). AFs were more toxic for the mitochondrial activity of the cells with EC_50_ values of 11.35 µg/mL and 30.52 µg/mL for AFB1 and AFB2, respectively. OTA was similarly toxic at the mitochondria and lysosomal levels, with EC_50_ values of 42.85 µg/mL and 43.95 µg/mL, respectively. DON and its metabolites presented a higher toxicity at the lysosomal level, with EC_50_ ranging from 43.95 to 61.61 µg/mL. Nivalenol and FBs were non-toxic for the fish cell line.

**Figure 1:**
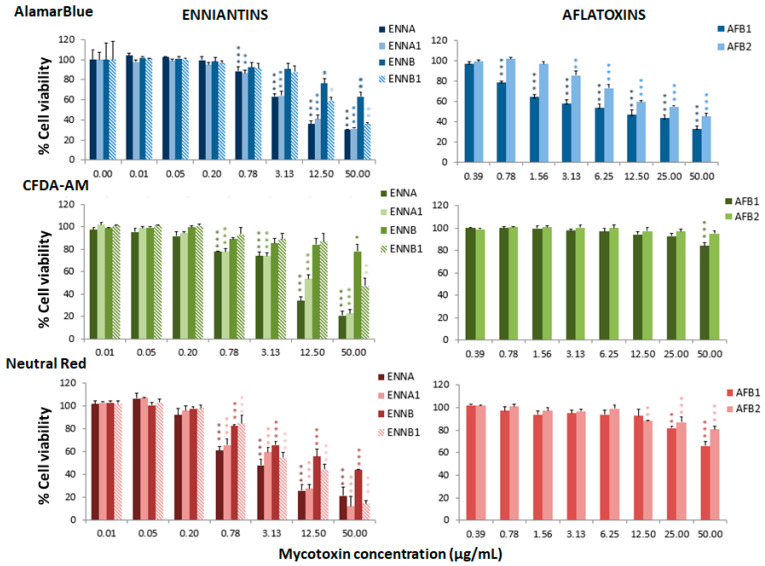
Percentage of cell viability with respect to the control group after 24 h of exposure of RTgill-W1 to enniatins or aflatoxins, expressed as mean ± SEM, *n* = 3. Significant differences with respect to the lowest concentration treated group: * (*p* < 0.05), ** (*p* < 0.01) and *** (*p* < 0.001); RMANOVA followed by Dunnett’s post-hoc test.

**Figure 2:**
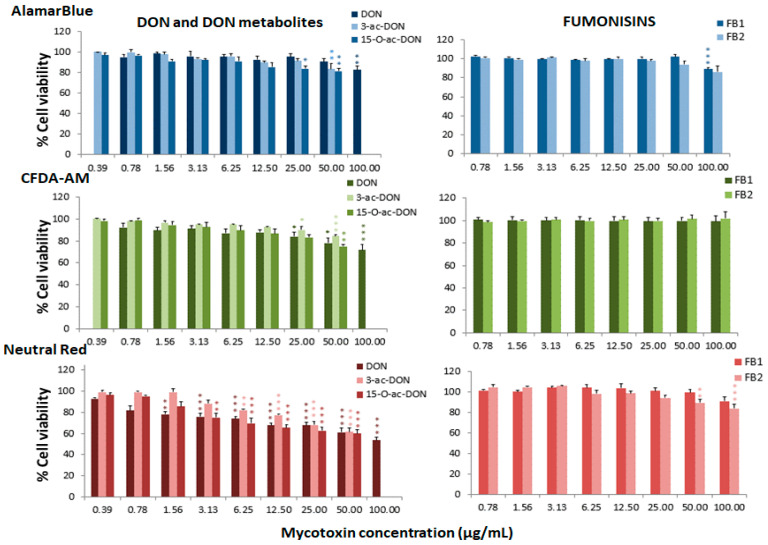
Percentage of cell viability with respect to the control group after 24 h of exposure of RTgill-W1 to DON, DON metabolites and fumonisins, expressed as mean ± SEM, *n* = 3. Significant differences with respect to the lowest concentration treated group: * (*p* < 0.05), ** (*p* < 0.01) and *** (*p* < 0.001); RMANOVA followed by Dunnett’s post-hoc test.

**Figure 3:**
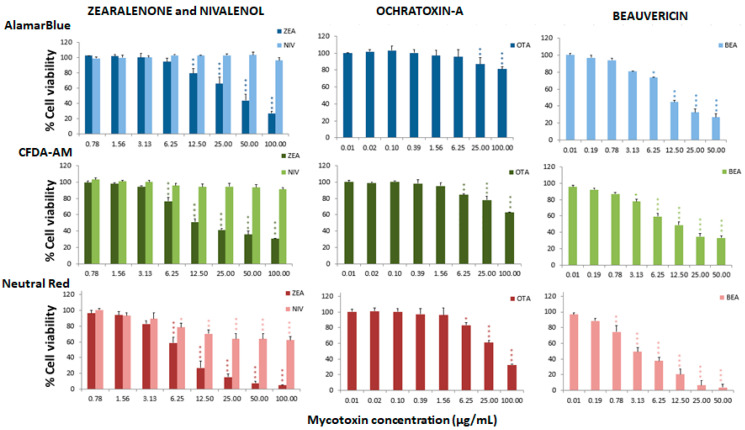
Percentage of cell viability with respect to the control group after 24 h of exposure of RTgill-W1 to ZEA, NIV, OTA and BEA, expressed as mean ± SEM, *n* = 3. Significant differences with respect to the lowest concentration treated group: * (*p* < 0.05), ** (*p* < 0.01) and *** (*p* < 0.001); RMANOVA followed by Dunnett’s post-hoc test.

Figure 1, Figure 2 and Figure 3 represent the cytotoxicity of the mycotoxins in the range of concentrations tested in the triple assay. For the ENNs group (Figure 1), regarding the alamarBlue assay, the lowest observed effect concentrations (LOEC) were 0.78 µg/mL for ENNA and ENNA1 (*p* < 0.001 and *p* < 0.01, respectively) and 12.50 µg/mL (*p* < 0.05) for ENNB and ENNB1. In the CFDA-AM assay, a LOEC value of 0.78 µg/mL (*p* < 0.001) was obtained for ENNA and ENNA1, and of 50 µg/mL for ENNB and ENNB1 (*p* < 0.05 and *p* < 0.01, respectively). In the NRU assay, a LOEC value of 0.78 µg/mL (*p* < 0.001) was observed for all the tested ENNs. For aflatoxins (Figure 1), the alamarBlue test revealed significant toxicity at very low concentrations with a LOEC value of 0.78 µg/mL for AFB1 (*p* < 0.001) and of 3.13 µg/mL for AFB2 (*p* < 0.01). Regarding the CFDA-AM assay, significant differences (*p* < 0.001) were only observed for AFB1, with LOEC corresponding to the highest concentration tested (50 µg/mL). Both AFB1 and AFB2 showed significant toxicity at the lysosomal level at 25 µg/mL and 12.5 µg/mL, respectively. With respect to the toxicity of DON and its metabolites 3-acetyl-DON and 15-O-acetyl-4-DON, the NRU assay was the most sensitive, with LOEC values of 1.56 µg/mL, 6.25 µg/mL and 3.13 µg/mL (*p* < 0.01), respectively (Figure 2). LOEC values of 100 µg/mL (*p* < 0.01) and 25 µg/mL (*p* < 0.05) were observed for DON with the alamarBlue and CFDA-AM assays, respectively. In the case of 3-acetyl-DON, LOEC values of 50 µg/mL (*p* < 0.01) and 25 µg/mL (*p* < 0.05) were recorded for the alamarBlue and CFDA-AM assays, respectively. For the metabolite 15-O-acetyl-4-DON, LOEC values of 25 µg/mL (*p* < 0.05) and 50 µg/mL (*p* < 0.01) were found with the alamarBlue and CFDA-AM test, respectively. FBs were the least toxic of all mycotoxins tested (Figure 2). FB1 showed significant toxicity only at mitochondrial level, with a LOEC value of 100 µg/mL (*p* < 0.001) related to the alamarBlue assay, while FB2 only showed a significant effect at lysosomal level with a LOEC value of 50 µg/mL (*p* < 0.01) evidenced by the NRU assay. NIV only showed toxicity at lysosomal membrane integrity level (NRU) with LOEC values of 6.25 µg/mL (*p* < 0.05) (Figure 3). In contrast, ZEN, OTA and BEA presented marked toxicity regarding the three different endpoints of cell metabolism (Figure 3). A LOEC value of 12.5 µg/mL (*p* < 0.01) was found for ZEN by the alamarBlue assay, while a LOEC of 6.25 µg/mL (*p* < 0.001) was observed in both CFDA-AM and NRU assays. OTA showed a significant effect with the alamarBlue assay with a LOEC value of 25 µg/mL (*p* < 0.01) and also with the CFDA-AM (*p* < 0.01) and NRU assays, with a LOEC value of 6.25 µg/mL (*p* < 0.01 and *p* < 0.05, respectively). Finally, the observed toxicity for BEA was significant in all three assays, with LOECs of 0.78 µg/mL (*p* < 0.05) for alamarBlue, 3.13 µg/mL (*p* < 0.05) for CFDA-AM and 0.78 µg/mL (*p* < 0.01) for NRU.

## 3. Discussion

The growing use of plant-based material in the manufacture of aquafeeds has led to new concerns in aquaculture regarding mycotoxin contaminations. This study was designed to investigate the cytotoxic effects produced by fifteen common mycotoxins, reported as aquafeed contaminants, in the fish cell line RTgill-W1 which is recommended in the prediction of the acute toxicity of chemical compounds in fish [14,15,16]. This gill cell line from rainbow trout was chosen by Tanneberger et al. [14], as the gill epithelia of fish are the primary uptake sites of water contaminants. These authors proposed the use of fish cell lines instead of mammalian cells for testing the toxicity of aquatic contaminants, because they reflect better than mammalian cells the properties of fish. In fact, chemicals can be applied to fish cells at the temperatures to which fish would be exposed [14]. EC_50_ values derived from the RTgill-W1 cell line assay were demonstrated to be in excellent agreement with lethal concentrations (LC_50_ values) determined in acute toxicity tests in fish for a wide range of chemicals. Some exceptions were described. These exceptions were reported for neurotoxic chemicals and for some chemicals of which the transformation products were more toxic, despite a proven ability of the cell line to biotransform chemicals [14,16]. The applicability of this in vitro system to the study of the acute toxicity of chemicals administered to fish via diet remains to be investigated. However, previous studies performed in our laboratory [17,18] indicated no big differences in cytotoxicity observed among fish cell lines.

Our results showed that the RTgill-W1 fish cell line was sensitive to most of the mycotoxins tested at the concentrations used. A ranking of toxicities could be established: ENNA = ENNA1 = BEA > ENNB1 > ZEA > AFB1 > ENNB > AFB2 > OTA = DON = 15-O-ac-DON > 3-ac-DON. NIV and FBs showed EC_50_ > 100 µg/mL, indicating they did not induce an acute effect in the cells. We observed that lysosomal membrane integrity, assessed by NRU assay, was the most sensitive endpoint for predicting cytotoxic effects of mycotoxins in vitro. Only AFBs were more toxic at the mitochondrial level.

The ENN mycotoxins were shown to be highly toxic at the lysosomal level for the RTgill-W1 fish cell line, with EC_50_ values lower than 5 µg/mL for ENNA, ENNA1 and ENNB1, and of 17 µg/mL for ENNB. Our results accorded with the toxicity reported for ENNs in zebrafish larvae and embryos, where effects were observed at low concentrations, and stronger effects were observed for ENNA than for ENNB [19]. In a recent review of in vitro effects of ENNs conducted on human and mammalian intestinal cell models, mitochondrial and lysosomal alterations were reported, as well as ROS production and lipid peroxidation at low concentrations [20]. Olleik et al. [21] observed that, although ENN and BEA have a limited hemolytic effect, they are toxic at low doses to various human cell lines. Their study was carried out in BEAS-2B (human normal airway cells), Caco-2 (human intestinal cell line), HEK (human normal keratinocytes), HEPG2 (human liver cell line), HUVEC (human normal vascular endothelial cells) and N87 (human gastric cell line) cells, showing that the effect, and also the ranking of toxicity for ENNs and BEA, are dependent on the cell type. The study of Prosperini et al. [22] on the toxicity of ENNs in Caco-2 cells confirms the order of ENN cytotoxicity found in our study: ENNA > ENNA1 > ENNB1 > ENNB.

BEA showed a high toxicity for the three endpoints of toxicity studied, with EC_50_ values between 3 and 13 µg/mL. The toxic effect of BEA was mainly exerted at the lysosomal level. The cytotoxicity of BEA was also reported in the fish cell lines PLHC-1 and RTH-149 and in the mammalian cell line H4IIE [23]. In this study, high toxicities were also observed with EC_50_ values ranging from 1.7 to 13.6 µg/mL. At plasma and lysosomal membrane levels, rat cells were shown to be more sensitive than fish cells whereas at mitochondrial level the H4IIE and PLHC-1 cell lines showed no significant differences. The toxicity of BEA was also studied by Olleik et al. [21] in different nucleated human cell lines, where BEA gave EC_50_ values ranging from 5.7 to 43.7 µM after 48 h exposure as evidenced by the alamarBlue assay. An MTT assay was also used in SH-SY5Y human neuronal cells [24] to assess BEA toxicity at the mitochondrial level. An EC_50_ value of 2.5 µM (equivalent to 1.96 µg/mL) could only be determined after 72 h of exposure. The cytotoxic effect of BEA in Vero cells using MTT and NRU assays over 24, 48 and 72 h was studied by Ruiz et al. [25]. EC_50_ values ranging from 6.25 µM to 10.02 µM (equivalent to 4.8 µg/mL to 7.87 µg/mL) and from 6.77 µM to 11.08 µM (equivalent to 5.3 µg/mL to 8.69 µg/mL) for the MTT and NRU assays, respectively, were reported.

The effects of ZEA after 24 h exposure showed a high cytotoxicity mainly at the lysosomal level (EC_50_ of 8 µg/mL). The EC_50_ values at the plasma membrane and mitochondrial levels were of 23 and 43 µg/mL, respectively. These results are in agreement with the results obtained by Pietsch et al. [26] in five different fish cell lines including the RTgill-W1. The NR assay appeared to be a more sensitive endpoint than the MTT, with EC_50_ values of between 2.8 and 8.9 µg/mL. Similar results were also reported in the study of Zhou et al. [13] in the BF-2 fish cell line (bluegill fry, *Lepomis macrochirus*) where viability curves were determined by the resazurin assay. In this study, the EC_50_ value was 170.24 µM after 48 h of exposure (equivalent to 54.20 µg/mL). These authors also studied the effect of ZEA in zebrafish larvae after a 48 h exposure. The LD_50_ was 13.83 µM (equivalent to 4.40 µg/mL) which indicated a higher sensitivity of this organism to ZEA. The toxic effects and accumulation of ZEA were also studied in different fish species, revealing accumulation mainly in the ovaries [27], and toxic effects on the immune system and at reproductive level, such as decreases in the reproductive performance of the next generation and in spawning frequency [4,28].

The toxic effect of AFs was produced mainly at the mitochondrial level. AFB1 was three times more toxic than AFB2, with an EC_50_ of 11 in comparison with 30 µg/mL for AFB2 in our study. Additional studies have shown that very low concentrations (<1 µg/mL) of AFB1 exert high toxic effects in the primary hepatocytes of *Cyprinus carpio* and sea bream fish [29,30]. AFB1 also presents a high toxicity in mammalian cell lines. In the hepatic cell line BFH12 from cattle, EC_50_ values of 6.34 µM (1.98 µg/mL) and 5.15 µM (1.60 µg/mL) were obtained after 48 h and 72 h exposure to AFB1 [31]. AFB1 was also highly toxic for the neuroblastoma cell line IMR-32 with EC_50_ values of 6.18 µg/mL after 24 h exposure [32]. A recent study carried out in the human cells NH4-SV40LT [33] showed a decreased cell proliferation of 41% at 50 µM (15.61 µg/mL) of AFB1. This study verified that AFB1 treatment induced mitochondrial calcium changes in humans. The mechanism of toxicity for AFB1 explained in this article could explain the higher sensitivity of the alamarBlue assay obtained in our study for AFB1. The in vivo toxicity of AFB1 in different fish species has been extensively studied, revealing negative effects on animal health [34,35,36,37,38]. The acute exposure induced aflatoxicosis and the accumulation of AFB1 in the liver, muscle and ovaries of different fish species after long-term exposure. Aflatoxicosis was associated with poor growth rates, lack of weight gain, pale gills, reduced survival rate and abnormal behavior. AFB1 toxicity was also associated with decreased growth, histopathological alterations and immune system effects [4,35,39]. Rainbow trout are extremely sensitive to AFB1. Diets containing AFB1 at concentrations lower than 0.02 mg/kg induced tumors after 12 months of exposure [40,41].

OTA was shown to be less toxic than the previous mycotoxins, with EC_50_ values of around 43 µg/mL at mitochondrial and lysosomal level. No cytotoxicity was observed at plasma membrane level. It is important to be aware of the high affinity of OTA for proteins, particularly serum albumins [42]. In our study, the culture medium was supplemented with 10% fetal bovine serum (FBS). Thus, the effects of OTA may have been lower than if it had been tested in a serum-free medium. The cytotoxicity of OTA was previously assayed using the triple assay in two fish cell lines (PLHC-1 and RTH-149) and in a rat cell line (H4IIE) exposing cells to a range of concentrations up to 40.4 µg/mL [23]. EC_50_ values higher than 40.4 µg/mL were reported at the three endpoints studied in the RTH-149 fish cell line. In contrast, OTA was toxic at the lysosomal level in the PLHC-1 and H4IIE cell lines, with EC_50_ values of 5.5 and 21.6 µg/mL, respectively. EC_50_ values higher than 100 µM (equivalent to 40.40 µg/mL) were found in LLC-PK1 (pig kidney epithelial cells) at metabolic and lysosomal levels using the MTT and NRU assay, respectively [43]. Other authors reported EC_50_ values of 30.28 µg/mL and 14.24 µg/mL in the human cell line HepG2 at the mitochondrial level [44,45]. According to the review by Oliveira and Vasconcelos [4], fish appear to be particularly sensitive to this mycotoxin with acute oral LC_50_ (96 h) of 0.28 mg/kg reported in adult sea bass (*Dicentrarchus labrax* L.). The loss of sensibility of the fish cell lines could be explained by the low availability of OTA to the cells due to its binding to the albumin.

DON and its metabolites only produced effects at the lysosomal level, EC_50_ ranging from 44 to 62 µg/mL. There are several studies on the cytotoxicity of DON in different mammalian and human cell lines, but only a few studies have been conducted in fish cell lines [13,23,46,47]. Within the studies carried out in fish cell lines, toxicity was not only reported at lysosomal level but also at other cellular levels, such as mitochondrial activity. In addition, the authors obtained lower EC_50_ values at all endpoints assayed and in all the tested cells. In the study carried out by Mayer et al. [46], the viability of RTgill-W1 was determined after treatment with DON with the NRU assay. It was observed that the viability of the cells was reduced by 52% after 48 h exposure at the highest concentration tested (40 µM equivalent to 11.85 µg/mL). Pietsch et al. [47] evaluated cell viability after 24 h of exposure of five different fish cell lines to DON by the MTT and NRU assays. Cell lines derived from rainbow trout (RTL-W1 and RTgill-W1) were more sensitive to DON than the cell lines derived from Atlantic salmon (SHK-1) and common carp (CCB). García-Herranz et al. [23] reported EC_50_ values of 1.52 µg/mL at the lysosomal level in the PLHC-1 cell lines, whereas EC_50_ values for RTH-149 could not be calculated at the highest concentration tested (29.60 µg/mL). Mayer et al. [46] studied the toxicity of DON in different mammalian cell lines. Cells derived from intestinal porcine epithelial cells (IPEC-1 and IPEC-J2) showed toxic effects at concentrations below 1 µg/mL. The cytotoxicity effect of DON in Vero cells has been evaluated by NR and MTT with EC_50_ values ranging from 5 to 11 µM (equivalent to 1.48–3.25 µg/mL) [25]. EC_50_ values of 0.60 µg/mL and 0.11µg/mL were reported in HepG2 and RAW 264.7, respectively [48]. To the best of our knowledge, the cytotoxicity of DON metabolites has not been investigated in fish cell lines and there are few articles focusing on their toxicity in mammalian cells. Results differed depending on the cell studied and the assay tested [49,50,51]. The acute in vivo toxicity of DON and its metabolites in fish has not been studied, and data concerning its toxicity are scarce. Hooft et al. [52] reported high sensitivity in rainbow trout fed with increasing levels of DON (0.3–2.6 mg/kg) for eight weeks. They reported feed refusal, reduction in feed conversion efficiency and reduction in weight gain and growth rate. Similar effects were reported by other authors in other fish species [4].

NIV and FBs did not present an acute effect in the fish cell line tested in our study. This result is in agreement with the results reported in a study on the fish cell line RTL-W1 for FB1 [53]. In this study, the toxic effect of FB1 on lysosomal activity was studied using the NRU assay, and the EC_50_ was 1746 μM (equivalent to 1260.31 µg/mL). These authors also reported EC_50_ values of 919 µM (equivalent to 663.36 µg/mL) in zebrafish embryos. In other mammalian and human cell lines, FB1 did not produce toxic effects at concentrations up to 100 µg/mL [53,54,55]. There are several studies on NIV in vitro cytotoxicity in mammalian cell lines, but to the best of our knowledge this work is the first conducted in a fish cell line. In the review by Zingales et al. [56] on cytotoxic effects reported in mammalian and human cell lines, NIV appears to alter cell proliferation, especially in tissues with high rates of cell turnover. High toxicities were reported, with EC_50_ values below 1 µM (equivalent to 0.30 µg/mL).

Thus, there is clearly a need for greater understanding of the consequences of the occurrence of mycotoxins in fish feeds, such as their toxicity and bioaccumulation in fish. Further research in this field is essential to find new ways to avoid and remove these toxic compounds, and to establish regulatory limits for acceptable levels of each type of mycotoxin in fish feeds.

## 4. Conclusions

For most of the mycotoxins tested (BEA, DON, 3-ac-DON, 15-O-ac-DON, ENNA, ENNA1, ENNB1, ENNB, NIV, OTA and ZEA), the most sensitive assay to evaluate cytotoxicity was the NRU, which indicated cell damage at lysosomal level. However, for AFBs the alamarBlue was the most sensitive assay, suggesting that these mycotoxins exert their toxic effects preferentially at mitochondrial level. Among the studied mycotoxins, ENNA, ENNA1, ENNB1, BEA and ZEA were the most highly toxic with EC_50_ values lower than 10 µg/mL, followed by ENNB and AFs. OTA and DON and its metabolites 3-ac-DON and 15-O-ac-DON were less toxic (EC_50_ around 50 µg/mL), whereas FBs and NIV were not toxic. The results indicated that most of the mycotoxins tested exert a highly acute effect in RTgill-W1 fish, emphasizing that mycotoxin occurrence in aquafeeds is a concern for both the aquaculture industry and for the consumers. The results point to the need for further assessments of the toxicity of mycotoxins ingested by fish through feed. Moreover, further research is needed to understand the bioaccumulation profile of mycotoxins in fish, and to find ways to remove these toxic compounds from feeds.

## 5. Materials and Methods

### 5.1. Reagents and Chemicals

Dimethyl sulfoxide (DMSO), methanol (≥99.9% purity), neutral red (NR), glacial acetic acid and all mycotoxins (ENNA, Cat. #: E9661; ENNA1, Cat. #: E5161; ENNB, Cat. #: E5411; ENNB1, Cat. #: E5286; AFB1, Cat. #: A6636; AFB2, Cat. #: A9887; FB1, Cat. #: F1147; FB2, Cat. #: F3771; ZEN, Cat. #: B7510; OTA, Cat. #: O1877; BEA, Cat. #: B7510; NIV, Cat. #: 32929; DON, Cat. #: 32943; 15-O-acetyl-4-DON, Cat. #: A1556; 3-acetyl-DON, Cat. #: A6166) were purchased from Sigma-Aldrich (Madrid, Spain). Ethanol was obtained from Panreac (Barcelona, Spain). 7-Hydroxy-3H-phenoxazin-3-one-10-oxide (alamarBlue, AB) and 5-carboxyfluorescein diacetate acetoxymethyl ester (CFDA-AM) were purchased from Invitrogen (Madrid, Spain). Leibovitz’s L-15 Medium (Cat. #11415-049) was obtained from Gibco (Madrid, Spain). Fetal bovine sera (FBS), penicillin/streptomycin (P/S, 10000 U/mL and 10 mg/mL respectively), ethylenediaminetetraacetic acid (EDTA) and 0.5% trypsin/0.02% EDTA were obtained from Lonza (Barcelona, Spain).

### 5.2. Preparation of Mycotoxins for Cytotoxicity Studies

The stock solutions of mycotoxins were prepared as follows: ENNA, ENNA1, AFB1 and AFB2 in DMSO at 2 mg/mL (2933 µM, 2994.59 µM, 6404.10 µM and 6363.35 µM, respectively); FB1 and FB2 in DMSO at 5 mg/mL (6934.81 µM and 7092.20 µM); ENNB and ENNB1 in DMSO at 8 mg/mL (12503.52 µM and 12235.22 µM); ZEN, NIV and OTA in DMSO at 10 mg/mL (31407.03 µM, 32018.44 µM and 24764.73 µM, respectively); 15-O-acetyl-4-DON and 3-acetyl-DON in methanol at 2.85 mg/mL (8423.23 µM and 8348.71 µM); DON in methanol at 11.1 mg/mL (37459.50 µM); and BEA in methanol at 5.5 mg/mL (7015.75 µM).

### 5.3. Cytotoxicity Studies

#### 5.3.1. Cell Culture and Exposure

An RTgill-W1 cell line was used to evaluate the cytotoxic effects associated with different mycotoxins. This cell line was obtained from the American Type Culture Collection (ATCC, CRL-2523™, Manassas, VA, USA) and cultured in 75 cm^2^ flasks at 20 °C, under a humidified atmosphere without CO_2_. Cells were grown in Leibovitz’s L-15 medium which contained L-glutamine and L-amino acids and which was supplemented with 10% FBS and 1% penicillin/streptomycin. Cells were split twice each week, using PBS/EDTA to wash and trypsin/EDTA mixture to detach the cells. To expose the cells to the mycotoxins, cells were seeded into transparent, flat-bottomed 96-well plates (Greiner Bio-One GmbH, Frickenhausen, Germany) by adding 100 µL of cell suspension (2.5 × 10^4^ cells/mL) per well. These were left overnight for cell attachment. Cells were then exposed for 24 h to serial dilutions of mycotoxins at the following concentration ranges: 0.012–50 µg/mL (0.019–78 µM) for ENNs, 0.012–50 µg/mL (0.01564 µM) for BEA, 0.012–100 µg/mL (0.03–248 µM) for OTA, 0.78–100 µg/mL (1.11–148 µM) for FB1 and FB2, 0.78–100 µg/mL (2.63–337 µM) for DON, 0.78–100 µg/mL (2.45–314 µM) for ZEN, 0.78–100 µg/mL (2.50–320 µM) for NIV, 0.39–50 µg/mL (1.25–160 µM) for AFB1 and AFB2 and 0.39–50 µg/mL (1.15–148 µM) for 3-acetyl-DON and 15-O-acetyl-4-DON. Cells were also exposed to serial dilutions of SDS as a positive control, and to the highest concentrations of solvent, either methanol or DMSO (0.08–10% *v*/*v*), to eliminate cytotoxic effects exerted by the vehicle. In addition, cells receiving only the medium were used as negative controls. Every mycotoxin concentration and the negative and solvent controls were tested in triplicate in each plate, and at least three independent experiments were carried out for each mycotoxin.

#### 5.3.2. Triple Assay: alamarBlue, CFDA-AM and NRU Assays

A triple assay consisting of the alamarBlue, CFDA-AM and NRU assays was performed on the RTgill-W1 (cell passages number 4–21) following the method described in Lammel et al. [57]. After mycotoxin exposure, the medium was removed and the cells were washed with PBS, before adding MEM containing 1% NEAA, 1.25 % (*v*/*v*) alamarBlue and 4 µM CFDA-AM to each well. Cells were incubated in this solution for 30 min in darkness under the culture conditions for the cell line. Afterwards, fluorescence intensity was measured at excitation/emission wavelengths of 532/590 nm and 485/535 nm for the alamarBlue and the CFDA-AM assay respectively. Cells were then washed with PBS and incubated in NR solution (0.03 mg/mL) for 1h. After a wash with PBS to remove excess of NR, the dye retained in viable cells was extracted with a solution of absolute ethanol:glacial acetic acid 1:1 (*v*/*v*). NR fluorescence was measured at excitation/emission wavelengths of 532/680 nm. All fluorescence measurements were performed in a Tecan Spark20 microplate reader (Tecan Group Ltd., Männedorf, Switzerland). Fluorescence readouts were corrected by subtracting the fluorescence intensity measured in cell-free wells, and normalized to the vehicle control and/or the cells receiving only the medium.

### 5.4. Statistical Analysis

All data were represented as the mean ± standard error of the mean (SEM) of three independent experiments performed in triplicate. All statistical analyses were performed using Sigma Plot (version 14.0, Systat Software, Inc., Chicago, IL, USA). The normality and homoscedasticity of all data were automatically checked with the program. The normality of the distribution and homogeneity of variance were confirmed with the Shapiro–Wilk test and Brown–Forsythe test, respectively. Significant differences among the groups treated with mycotoxins and the lowest concentration treated group, for which no effects were observed, were determined using a one-way repeated measures analysis of variance (RMANOVA, *p* < 0.05) followed by a Dunnett’s post-hoc test. The concentration needed to cause a 50% reduction in the effect with respect to the control group (EC_50_) was calculated from each dose–response curve obtained in each independent experiment using the Excel macro: REGTOX (Free Software Foundation, Inc., 59 Temple Place—Suite 330, Boston, MA 02111, USA) and the mean ± SEM (*n* = 3) was provided.

## Figures and Tables

**Table 1:** Mycotoxin EC_50_ concentrations expressed in µg/mL (mean ± SEM, *n* = 3) and in µM at the three different endpoints tested in the triple assay in RTgill-W1 after 24 h of exposure.

	EC_50_					
Mycotoxins	alamarBlue		CFDA-AM		NRU	
	µg/mL	µM	µg/mL	µM	µg/mL	µM
ENNA	8.44 ± 1.36	12.37	7.55 ± 1.01	11.07	2.89 ± 0.57	4.24
ENNA1	7.58 ± 1.25	11.34	11.19 ± 1.80	16.75	3.81 ± 0.95	5.70
ENNB	71.78 ± 16.56	112.19	>100	>156.29	17.03 ± 1.36	26.62
ENNB1	22.56 ± 3.10	34.50	50.50 ± 13.43	77.23	5.54 ± 0.66	8.47
BEA	13.05 ± 2.00	16.64	13.13 ± 2.19	16.75	3.01 ± 0.60	3.84
ZEN	43.03 ± 9.13	135.14	22.76 ± 1.84	71.48	8.02 ± 1.48	25.19
AFB1	11.35 ± 2.50	36.34	>100	>320.20	84.65 ± 14.08	269.33
AFB2	30.52 ± 1.72	97.10	>100	>318.17	>100	>318.17
OTA	42.85 ± 9.13	106.12	>100	>247.65	43.95 ± 10.61	108.84
DON	>100	>337.47	>100	>337.47	51.58 ± 5.08	174.07
3-ac-DON	>100	>292.94	>100	>292.94	61.61 ± 9.39	180.48
15-O-ac-DON	>100	>295.55	>100	>295.55	45.99 ± 10.34	135.92
NIV	>100	>320.18	>100	>320.18	>100	>320.18
FB1	>100	>138.69	>100	>138.69	>100	>138.69
FB2	>100	>141.84	>100	>141.84	>100	>141.84

## Data Availability

The data presented in this study are available in article here.
